# Comment on “5-Azacytidine Promotes an Inhibitory T-Cell Phenotype and Impairs Immune Mediated Antileukemic Activity”

**DOI:** 10.1155/2015/871641

**Published:** 2015-03-08

**Authors:** Thomas Mørch Frøsig, Sine Reker Hadrup

**Affiliations:** National Veterinary Institute, Technical University of Denmark, 1870 Frederiksberg, Denmark

With great interest we read the recent paper in Mediators of Inflammation by Thomas Stübig and colleagues [1] as the aim of this study was similar to the aim of a study we recently conducted [2], namely, to study the impact of the demethylating agent 5-Azacytidine on the immune system. The two studies, however, reached different conclusions, which as we will discuss below may originate from differences in the patient cohorts and the translation of data from* in vitro* analyses into* in vivo* effect. Thus, with the present commentary we would like to discuss the challenges in understanding the true effect of 5-Azacitidine on immune reactivity in cancer patients and how this may depend on the patient group analyzed. 5-Azacytidine was marketed (as Vidaza, Celgene Corporation, Boudry, Switzerland) after a phase III trial revealed it as the first drug prolonging overall survival in high-risk myelodysplastic syndrome (MDS) patients [3]. 5-Azacytidine is known to upregulate the expression of tumor suppressor genes [4], and it has been speculated to what extent it impacts the immune system, both directly and indirectly.

Stübig and coworkers analyzed the blood from healthy donors subjected to* in vitro* stimulation with 5-Azacytidine. They showed 5-Azacytidine-mediated inhibition of CD8 growth and killing capacity against a leukemic cell line, induction of CD4 regulatory T cells, reduction in proinflammatory Th1 cells, a shift in phenotype from memory to naïve for CD4 and CD8 T cells, and overexpression of the cell cycle inhibitor p15—in essence an inhibition of antileukemic immunity. These findings are interesting, but it should be noted that all analyses were done* in vitro* using blood from healthy donors and, moreover, all of these were performed with 5 or 20 *µ*M 5-Azacytidine. Conclusions related to the actual* in vivo* effect in cancer patients should be carefully drawn. We conducted* ex vivo* analyses using blood from a group of higher risk MDS and acute myeloid leukemia (AML) patients and did not detect the significant immune modulatory effects as observed by Stübig and coworkers* in vitro*. We obtained blood samples from seventeen patients diagnosed with MDS or AML before and after treatment with 5-Azacytidine at several time points for* ex vivo* investigation. We isolated CD8 T cells and CD34 myeloid blast cells (as a surrogate marker for the tumor cells) and were able to show that 5-Azacytidine treatment increased the T-cell mediated recognition of these by directly affecting the tumor cells, while the CD8 T cells were not affected. This effect may relate to 5-Azacytidine-mediated upregulation of cancer-testis antigens and/or MHC class I molecules, as has been described [4–7]. We were not able to correlate this directly due to a limited amount of cell material, but we also screened for a broad range of CD8 T-cell populations specific for cancer-testis antigens with MHC multimers and found a significant increase in the proportion of T cells recognizing these upon initiation of treatment. Further, we investigated the absolute numbers of the general populations of CD4 and CD8 T cells, regulatory CD4 T cells, and myeloid-derived suppressor cells and found no significant differences upon treatment with 5-Azacytidine, when comparing the level prior to treatment and at a late sample obtained at 4th–6th cycle. Expression of the regulatory T-cell marker FOXP3 has previously been shown to be strongly regulated by methylation* in vitro* [8] and* in vivo* in a transplantation setting [9], but the treatment did not increase the regulatory T-cell population in absolute numbers in our patients.

Thus, there seems to be a discrepancy between the* in vitro* assessments and the* in vivo* effect and further between different patient groups* in vivo*. Clinically the drug reaches a peak concentration of around 3 *µ*M when patients are treated subcutaneously with 75 mg/m^2^ [10] and is expected to reach 4 *µ*M upon* in vivo* treatment with 100 mg/m^2^ as was the dose used in both* in vivo* studies discussed here. The use of 5 *µ*M as the lowest concentration* in vitro* thus represents a 25% overdose while 20 *µ*M is out of range compared to the treatment level. Others have previously investigated the effect of 5-Azacytidine* in vitro* on the Natural Killer (NK) cells and found that 5-Azacytidine impairs NK cell reactivity* in vitro* [11, 12]. We confirmed this finding after 5-Azacytidine exposure at 2.5 and 5.0 *µ*M. The effect was, however, not as evident* in vivo* and we only noted a trend towards a decrease in the absolute numbers of NK cells along with a small, although significant, increase in NK cells with an inhibitory phenotype. Further, we found the* in vitro* impairment to be concentration-dependent, as we also conducted the experiment with the calculated 8-hour physiological concentration on 0.88 nM [10] and found no inhibition of NK cell reactivity. It is not known what factors differing between the* in vitro* and* in vivo* situations that are responsible for these differences, but our data indicates that the immunological effect of 5-Azacytidine is very sensitive to concentration changes and that* in vitro* analyses even at the physiological relevant concentration are not necessarily relevant for the* in vivo* situation.

Furthermore, the* in vivo* immune modulatory effect of 5-Azacytidine may vary depending on the patient group studied. Stübig and colleagues analyzed the* in vivo *effect of 5-Azacytidine treatment in three patients after allogeneic stem cell transplantation (alloSCT) and observed a tendency for immune modulation comparable to their* in vitro* studies, that is, less CD8 and more CD4 cells, a decrease in activated CD3^+^HLA-DR^+^ cells, and a shift from memory to naïve CD4 and CD8 T cells. The development of regulatory T cells was more dynamic but seemed to increase and this is in line with the previous data on 5-Azacytidine treatment upon alloSCT in a larger patient group [9]. These alloSCT patients were lymphodepleted and treated in a setting of ongoing immune reconstitution (at 66, 96, and 127 days after transplantation, resp.). Reconstitution of the innate immune cells, for example, the NK cells following alloSCT, is quite fast, but the adaptive immune cell populations are delayed, and the T cells are not fully reconstituted until years after the depletion (as reviewed in [13]). Thus it may be speculated that the immune cells under these circumstances are more susceptible to epigenetic manipulation than patients that are not lymphodepleted. In addition, 5-Azacytidine was administered in this situation to treat relapse or minimal residual disease after the transplantation, while the patients we treated had a heavy disease burden. Thus there seems to be a difference in the immune modulation sensitivity between these two patient groups (alloSCT-treated MDS patients versus higher risk MDS and AML patients). Therefore, the effect mediated by 5-Azacytidine should be carefully monitored in the patient group of interest prior to the design of an immune modulating therapeutic intervention. Responses to 5-Azacytidine are often delayed (e.g., [3]) and it has been established that immune modulators often have a slower onset than cytotoxic drugs [14]. We hence decided to use samples obtained from 4th to 6th cycle of treatment as the earliest endpoint; for part of our investigations we even extended the analyses to the blood sample obtained after 10th cycle to be able to measure the long-term effects of treatment. Stübig and coworkers, on the contrary, exclusively used samples obtained after the initial treatment cycles, which may reveal a different response profile.

With these reflections we hope to have convinced you that the immune modulatory effects of treatment with 5-Azacytidine are complex and dependent on the clinical setting. Hence, it seems that* in vitro* measurements of this treatment are suboptimal and should be carefully assessed in the patient group of interest, as different patient groups may respond differently to the epigenetic modulation conceived by 5-Azacytidine.
